# Transferrin (rs3811647) gene polymorphism in iron deficiency anemia

**DOI:** 10.1186/1755-8166-7-S1-P38

**Published:** 2014-01-21

**Authors:** K Sri Manjari, KSPS Teja, M Sujatha, A Jyothy, Pratibha Nallari, A Venkateshwari

**Affiliations:** 1 Institute of Genetics and Hospital for Genetic Diseases, Osmania University, Hyderabad; 2 Department of Genetics, Osmania University, Hyderabad

## Background

Iron-deficiency anemia (IDA) is the most common type of anemia, caused by inadequate iron availability for hemoglobin production due to the lack of dietary iron or insufficient uptake of iron. Transferrin (TF) exerts a crucial function in the maintenance of systematic iron homeostasis. The expression of the TF gene is controlled by transcriptional mechanism, although little is known about its influence on IDA. Hence, the aim of the current investigation was to determine the functional polymorphism (rs3811647) of TF gene in iron deficiency anemia.

## Material and methods

A total of 207 school children of age from 12-16 years were selected from Government High School Seetaphalmandi, Secunderabad, Andhra Pradesh and screened for iron deficiency. Out of which 70 school children had iron deficiency anemia with hemoglobin levels less than 11.5 g/dl and 137 were normal. Demographic details and blood samples were obtained from all the subjects. Genotyping was carried out by tetra-primer ARMS PCR followed by agarose gel electrophoresis, and appropriate statistical analysis.

## Results

The genotype distribution of TF (rs3811647) region were 4.3% (AA), 81.4% (AG) and 14.3% (GG) in iron deficient children compared to 8% (AA), 72.3% (AG), and 19.7% (GG) in normal children. No significant variation was observed with respect to the allelic distribution [OD = 1.035 (0.67 – 1.59), p = 0.917] in the IDA group when compared to normal group.

## Conclusions

There was no significant association of the TF (rs3811647) gene polymorphism with iron deficiency anemia. Thus, our study highlights the importance of other genetic variants influencing the outcome of iron deficiency anemia. However, larger samples have to be analyzed to confirm the same.

